# Real-World Two-Year Outcomes of Diffractive Implantable Phakic Contact Lenses in Presbyopic Myopes: Functional Vision, Anatomical Findings, and Environmental Context

**DOI:** 10.3390/medicina62061019

**Published:** 2026-05-25

**Authors:** David Pérez-Silguero, Miguel Ángel Pérez-Silguero, Pablo Encinas-Pisa, Maria Mayoral-Álvarez, Alonso Verbo Gil, Sara Perez-Silguero Jimenez, Inmaculada Bernal-Blasco

**Affiliations:** 1 Miranza Clínica Pérez Silguero—Ophthalmology Service Hospital San José, 35003 Las Palmas de Gran Canaria, Spain; 2 Miranza Clínica Pérez Silguero—Ophthalmology Service Hospital La Paloma, 35003 Las Palmas de Gran Canaria, Spain; psilgueroma@gmail.com; 3 Miranza Clínica Pérez Silguero, 35003 Las Palmas de Gran Canaria, Spain; pablo.encinas@miranza.es (P.E.-P.); maria.mayoral@miranza.es (M.M.-Á.); alonso.verbo@miranza.es (A.V.G.); 4 Hospital General de Fuenlabrada, 28942 Madrid, Spain; sarapsilguero@gmail.com; 5 Centro de Salud Cuevas Torres, 35012 Las Palmas de Gran Canaria, Spain; miguel.perez.silguero@miranza.es

**Keywords:** diffractive implantable phakic contact lens, IPCL, presbyopia correction, presbyopic myopia, defocus curve, patient-reported outcomes, central vault, phakic intraocular lens safety, real-world visual performance, environmental exposure

## Abstract

*Background and Objectives*: To evaluate two-year functional, anatomical, and patient-reported outcomes after bilateral implantation of a diffractive implantable phakic contact lens (IPCL) for presbyopia correction in myopic patients within a high–solar-radiation Atlantic island environment. *Methods:* This retrospective observational study included 11 presbyopic myopic patients aged 40–50 years (22 eyes) who underwent bilateral diffractive IPCL implantation and completed a 2-year follow-up. Monocular defocus curves were recorded from +3.0 to −5.0 D and converted to logMAR. Functional visual range and area under the defocus curve (AUC) were calculated. Anatomical stability was assessed by central vault, pupil diameter, and crystalline lens rise measurements. Safety evaluation included slit-lamp examination for crystalline lens transparency and corneal integrity. Patient-reported outcomes were measured using the Quality of Vision (QoV) and Catquest-9SF questionnaires. Environmental parameters during implantation and follow-up were characterized using regional meteorological data. *Results:* Mean visual acuity remained ≤0.14 logMAR up to −2.5 D of defocus, with functional vision (≤0.2 logMAR) extending to approximately −3.0 D in about half of the eyes. Median vault at 2 years was 546 µm (IQR 361.5–667.3 µm). No cases of clinically significant cataract or corneal compromise were observed. QoV scores were low (1.41 ± 0.43) and Catquest-9SF scores high (3.70 ± 0.18), with no strong correlations between subjective and anatomical metrics. Visual performance remained stable within a consistent high-solar-radiation and atmospheric light-scattering environment. *Conclusions:* Diffractive IPCL implantation was associated with stable anatomical positioning, sustained functional visual performance across distances, and favorable patient-reported outcomes at 2 years. Within a consistent real-world environmental context, these findings provide a descriptive framework for understanding diffractive IPCL performance, while larger prospective studies are warranted.

## 1. Introduction

The surgical correction of myopia in presbyopic patients remains a clinical challenge, particularly in individuals with high myopia or anatomical features that increase the risk of retinal complications [1,2,3,4]. In this population, corneal refractive surgery and refractive lens exchange with multifocal intraocular lenses may be limited by safety concerns, predictability issues, or the desire to preserve the crystalline lens. As a result, lens-based but lens-preserving approaches have gained increasing interest for the simultaneous management of myopia and presbyopia [5,6].

Diffractive implantable phakic contact lenses (IPCLs; Care Group, India) have emerged as a novel concept in presbyopia management, combining the advantages of posterior chamber phakic lenses with diffractive optics designed to extend functional vision across distances. Early clinical experiences with diffractive phakic lenses have demonstrated promising outcomes in terms of distance and near visual performance, patient satisfaction, and anatomical compatibility. Initial reports have described encouraging short-term functional results and acceptable safety profiles, while more recent studies have begun to document medium- and long-term stability, particularly regarding vault behavior and anatomical positioning [6,7,8,9,10,11]. Nevertheless, the available literature remains limited, with relatively few studies providing detailed defocus curve analysis, long-term follow-up, or integrated evaluation of patient-reported outcomes.

In addition to optical design and anatomical compatibility, real-world visual performance is influenced by environmental visual demands, as variations in lighting conditions and atmospheric light scattering have been shown to significantly alter conscious contrast perception and visual task performance [12,13,14]. Most published reports on diffractive phakic lenses have been conducted under controlled clinical conditions, without considering the potential impact of environmental exposure on functional vision and subjective visual quality. Gran Canaria represents a distinctive ecological setting characterized by high annual solar radiation, persistent trade winds, marine aerosol exposure, and periodic Saharan dust events (“calima”), all of which can increase atmospheric light scattering and influence contrast perception under everyday conditions [15,16]. The island’s limited distance to the coastline and the predominance of outdoor daily activities result in continuous exposure to high-luminance environments and variable atmospheric transparency, providing a unique real-world framework for evaluating visual technologies.

The present study was designed to provide a comprehensive functional and ecological assessment of a diffractive implantable phakic contact lens for presbyopic myopic patients with a minimum follow-up of two years. Specifically, the study aimed to evaluate visual performance across distances using monocular defocus curves, assess anatomical stability and positioning parameters, and analyze patient-reported visual quality and satisfaction within a consistent high-exposure Atlantic environment.

## 2. Materials and Methods

### 2.1. Study Design and Setting

This observational retrospective study was conducted at Miranza Pérez Silguero Ophthalmology Center (Las Palmas de Gran Canaria, Spain). The study evaluated clinical, functional, anatomical, and patient-reported outcomes in presbyopic myopic patients who underwent bilateral implantation of a diffractive implantable phakic contact lens (IPCL) for simultaneous correction of myopia and presbyopia.

All procedures adhered to the tenets of the Declaration of Helsinki. Data were obtained exclusively from clinical records as part of routine clinical care. The study protocol was reviewed by the institutional research ethics committee (Comité de Ética de la Investigación con medicamentos del Instituto de Microcirugía Ocular; COD IMO 250509-289; 29 July 2025) and, due to its retrospective design and use of anonymized data, the requirement for informed consent was waived. The study was reported in accordance with the STROBE (Strengthening the Reporting of Observational Studies in Epidemiology) guidelines for observational studies.

### 2.2. Participants

The study included presbyopic myopic patients aged 40–50 years who underwent bilateral implantation of a diffractive IPCL between January 2020 and July 2023 and had a minimum postoperative follow-up of two years.

Inclusion criteria comprised availability of complete preoperative and 2-year postoperative clinical data, including visual performance assessment and patient-reported outcome measures. Exclusion criteria included ocular or systemic conditions that could affect visual performance, incomplete clinical records, or use of neuroactive medications that might interfere with subjective visual perception.

### 2.3. Surgical Procedure and Implanted Lens

All eyes underwent implantation of a posterior chamber diffractive implantable phakic contact lens (IPCL, Care Group, India). Lens power and toricity were selected according to standard manufacturer guidelines and individual refractive and anatomical parameters. Near addition (+2.5 or +3.0 D in this cohort) was determined based on both manufacturer recommendations and an original near addition strategy, taking into account patient age, degree of myopia, and subjective tolerance. In all cases, the goal was to balance functional near performance while preserving distance and intermediate vision. All procedures were performed by an experienced anterior segment surgeon under standard aseptic conditions. Postoperative management followed routine clinical protocols.

### 2.4. Near Addition Selection Strategy

Near addition power for the diffractive IPCL was not determined based on the age-equivalent spectacle addition typically prescribed for presbyopia. Instead, a functional tolerance–based strategy was used with the objective of selecting the highest addition that maintained comfortable binocular vision while providing a margin for future presbyopic progression.

The selection process followed a structured sequence. First, monocular distance refraction was optimized in each eye. Second, once best-corrected monocular distance visual acuity was achieved, near vision testing was performed binocularly at the patient’s preferred reading distance rather than at a fixed standardized distance, in order to reflect habitual visual behavior.

Third, incremental positive lenses were then introduced binocularly, starting at +1.00 D and progressively increasing (+1.50 D, +2.00 D, +2.25 D, and +2.50 D). The addition was increased until the patient first reported subjective visual discomfort or blur during sustained near viewing. From that point, the addition was adjusted in small increments above and below the threshold to identify the level that provided the best balance between clarity and sustained visual comfort.

The final selected addition corresponded to the highest addition that remained subjectively comfortable for the patient under these conditions. To ensure consistency, the entire assessment was repeated on a second visit, and the selected addition was confirmed only when both evaluations yielded concordant results. In all cases, the same addition power was implanted in both eyes. This individualized approach incorporated each patient’s habitual reading distance and visual demands, allowing customization according to daily functional needs.

### 2.5. Clinical and Anatomical Assessment

Two visits were considered for analysis: preoperative baseline visit and postoperative follow-up at 2 years. As part of routine postoperative clinical follow-up, the transparency of the crystalline lens and cornea was systematically evaluated at each visit using slit-lamp biomicroscopy. Particular attention was paid to early signs of anterior subcapsular opacification, lens densitometry changes, or corneal transparency alterations potentially associated with endothelial compromise. The presence or absence of cataract formation was specifically documented in the two-year clinical registry for all patients included in the study. From clinical records, the following variables were extracted: manifest refraction and visual acuity; dynamic pupil diameter under photopic and scotopic conditions; central vault (µm) measured using anterior segment optical coherence tomography (Anterion, Heidelberg Engineering, Germany); and crystalline lens rise measured using anterior segment imaging.

Vault measurements were obtained under controlled illumination conditions. Dynamic pupil measurements were obtained using a corneal topography system with infrared pupillometry (Keratograph 5M, Oculus, Wetzlar, Germany). Crystalline lens rise was defined as the perpendicular distance between the anterior crystalline lens surface and the horizontal line connecting the iridocorneal angles. All defocus measurements were performed under standardized photopic illumination to minimize variability related to pupil size and testing conditions.

### 2.6. Defocus Curve Assessment (Primary Outcome)

Monocular defocus curves were obtained at the 2-year visit using best distance correction under standardized photopic conditions. Visual acuity was measured in decimal notation at defocus levels from +3.0 to −5.0 diopters in 0.5-D steps.

For statistical analysis, all visual acuity values were converted to logarithm of the minimum angle of resolution (logMAR). Defocus curves were analyzed at the eye level. To provide a global metric of functional performance, the area under the defocus curve (AUC) was calculated for each eye in logMAR·diopter units using the trapezoidal rule. Functional visual range was defined as the continuous interval of defocus values maintaining visual acuity ≤0.2 logMAR. This threshold was selected based on commonly adopted criteria in defocus curve analysis and presbyopia-correcting intraocular lens studies, where this level is considered to represent clinically useful visual acuity for daily activities [17,18].

### 2.7. Patient-Reported Outcomes

Subjective visual quality and functional satisfaction were assessed at the 2-year visit using two previously validated patient-reported outcome measures: the Quality of Vision (QoV) questionnaire [19] and the Catquest-9SF instrument [20]. The QoV questionnaire evaluates the frequency, severity, and bothersomeness of visual symptoms such as glare, halos, starbursts, hazy vision, and visual fluctuations using a 4-point ordinal scale, where lower scores indicate fewer symptoms and better perceived visual quality. The Catquest-9SF assesses perceived visual disability, independence from spectacles, and overall satisfaction, with scores ranging from 1 to 4 and higher values indicating better perceived visual function. PROMs were analyzed at the patient level.

Patients were shown standardized visual representations of common dysphotopsia phenomena (glare, halos, and starbursts) and asked to identify the closest match to their visual perception and estimate relative symptom size.

Patient-reported outcome measures were obtained as part of the clinic’s routine quality-of-care assessment protocol. Patients were invited to complete the QoV and Catquest-9SF questionnaires voluntarily upon arrival for their scheduled postoperative visit, prior to clinical examination and without physician influence. Questionnaires were administered and collected by trained auxiliary staff as part of standard institutional practice.

### 2.8. Environmental and Geographic Context

To contextualize real-world visual performance, environmental conditions of Gran Canaria were characterized. All patients resided in Las Palmas de Gran Canaria or surrounding metropolitan areas, representing a subtropical Atlantic island environment with year-round outdoor visual exposure.

Climatic data corresponding to the study period were obtained from the Spanish State Meteorological Agency (AEMET) using the Gran Canaria Airport meteorological station (Gando), which provides standardized long-term environmental monitoring representative of the island’s coastal urban population.

For each year of implantation and corresponding two-year follow-up period, the following environmental parameters were recorded: Mean annual temperature (°C), Mean relative humidity (%), Mean wind speed (km/h), Number of days with Saharan dust events (calima), Mean annual solar radiation (when available).

These variables were used to characterize the environmental visual demands experienced by the cohort during both implantation and long-term postoperative periods. Environmental parameters were analyzed descriptively and explored for potential associations with patient-reported visual quality outcomes.

### 2.9. Statistical Analysis

Continuous variables were described using mean ± standard deviation or median and interquartile range according to data distribution and normality. Categorical variables were expressed as counts and percentages. Normality of continuous variables was assessed using the Shapiro–Wilk test.

Given the inclusion of both eyes from the same patient, analyses involving eye-level variables accounted for within-subject correlation. When appropriate, mixed-effects models with patient as a random effect were used. For analyses involving patient-reported outcome measures (PROMs), eye-level parameters were averaged per patient to obtain a single representative value.

Eye-level analyses were performed for optical and anatomical parameters, accounting for within-subject correlation, whereas patient-reported outcomes were analyzed at the patient level. For correlation analyses, eye-level variables were averaged per patient to avoid pseudo-replication.

Associations between anatomical parameters, functional visual performance metrics, and PROMs were evaluated using Spearman correlation coefficients. A two-sided *p* value < 0.05 was considered statistically significant. Correlation analyses were interpreted as exploratory.

In addition to predefined primary and secondary analyses, exploratory assessments were performed to evaluate potential associations between postoperative vault and selected lens-related variables. Specifically, correlations between central vault and absolute spherical power as well as near addition were explored using Spearman’s rank correlation coefficient. Differences in vault between toric and non-toric IPCL models were evaluated using the Mann–Whitney U test. These analyses were considered exploratory and hypothesis-generating and were interpreted descriptively without adjustment for multiple comparisons. Statistical analysis was performed using SPSS (version 27.0; IBM Corp., Armonk, NY, USA).

## 3. Results

### 3.1. Study Population and Implant Characteristics

A total of 11 presbyopic myopic patients (seven women and four men) who underwent bilateral implantation of a diffractive implantable phakic contact lens (IPCL) were included, yielding 22 eyes with a minimum postoperative follow-up of two years. The mean age at surgery was 44.1 ± 3.6 years (range 41–50). The mean spherical power of the implanted lenses was −7.16 ± 3.55 D (range: −14.0 to −2.5 D). The near addition ranged from +2.5 D to +3.0 D, with a mean addition of +2.66 ± 0.24 D. Addition power was +2.5 D in 15 eyes (68.2%) and +3.0 D in 7 eyes (31.8%). A toric model was implanted in 7 eyes (31.8%). Table 1 shows the lens characteristics.

### 3.2. Functional Visual Performance: Monocular Defocus Curve

Monocular defocus curves were obtained from +3.0 to −5.0 diopters in 0.5-D steps. Visual acuity was recorded in decimal format and subsequently converted to logMAR for statistical analysis. The resulting defocus profile demonstrated stable visual performance from distance through intermediate and functional near ranges, with the expected decline at higher accommodative demands.

At plano (0.0 D), mean visual acuity was 0.05 ± 0.20 logMAR. Visual performance remained favorable at intermediate defocus levels, with mean values of 0.11 ± 0.20 logMAR at −1.5 D and 0.14 ± 0.22 logMAR at −2.5 D, corresponding to functional intermediate and near distances. A gradual reduction in acuity was observed beyond −3.0 D, where mean visual acuity reached 0.22 ± 0.27 logMAR, followed by a more pronounced decline at higher near demands, with mean values of 0.40 ± 0.38 logMAR at −4.0 D and 0.62 ± 0.47 logMAR at −5.0 D.

**Figure 1:**
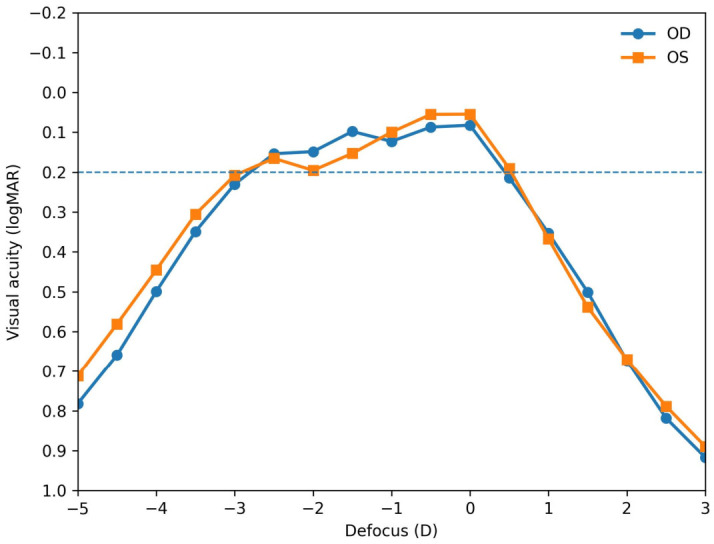
Monocular defocus curve at 2 years after bilateral diffractive IPCL implantation.

Overall, these findings indicate a broad plateau of high visual performance extending from distance to approximately −2.5 D of defocus, with progressive reduction beyond −3.0 D, as illustrated in Figure 1.

### 3.3. Functional Range

Using the conventional functional threshold of ≤0.2 logMAR, the lower boundary of functional vision clustered around −3.0 D. At −3.0 D, approximately 50% of eyes maintained functional visual acuity, decreasing to 45% at −3.5 D.

At the eye level, the median functional range extended from 0.0 D to −3.0 D, with interquartile limits between +0.5 D and −3.5 D. Global visual performance for each eye was summarized using the AUC, expressed in logMAR·D units, which showed inter-individual variability with a mean value of 2.87 ± 0.91 logMAR·D (range: 1.51–5.24). Lower AUC values were associated with broader functional visual ranges and more homogeneous performance across defocus levels.

### 3.4. Safety, Anatomical Stability, and Positioning Parameters at 2 Years

No cases of clinically significant cataract formation were identified in the clinical records at the two-year follow-up. Slit-lamp evaluation confirmed maintained crystalline lens transparency in all eyes. Similarly, no corneal opacities, edema, or clinically detectable alterations suggestive of endothelial decompensation were observed during routine postoperative examinations.

**Figure 2:**
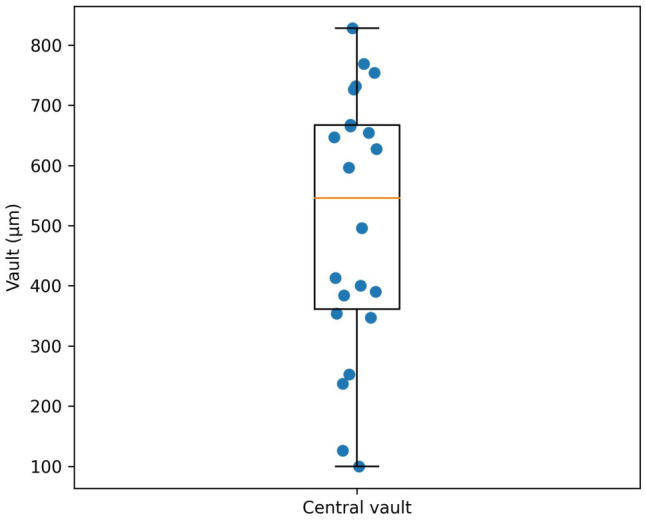
Postoperative central vault distribution at two-year follow-up. Box-and-whisker plot showing the distribution of central vault values (µm) measured two years after bilateral implantation of the diffractive IPCL. The central orange line represents the median value, the box indicates the interquartile range, whiskers represent the data range, and blue circles represent individual eye measurements.

All eyes maintained clear corneas and stable anterior segment findings throughout the follow-up period. At the two-year postoperative evaluation, the median central vault was 546 µm (IQR: 361.5–667.3 µm; range: 100–828 µm), indicating stable lens positioning within clinically acceptable limits throughout the cohort. Postoperative pupil diameter measurements demonstrated a median photopic pupil size of 4.4 mm (range: 2.9–5.8 mm) and a median scotopic pupil size of 5.15 mm (range: 2.4–7.4 mm). The distribution of vault values across the cohort is illustrated in Figure 2.

### 3.5. Patient-Reported Outcomes: Quality of Vision and Satisfaction

**Figure 3:**
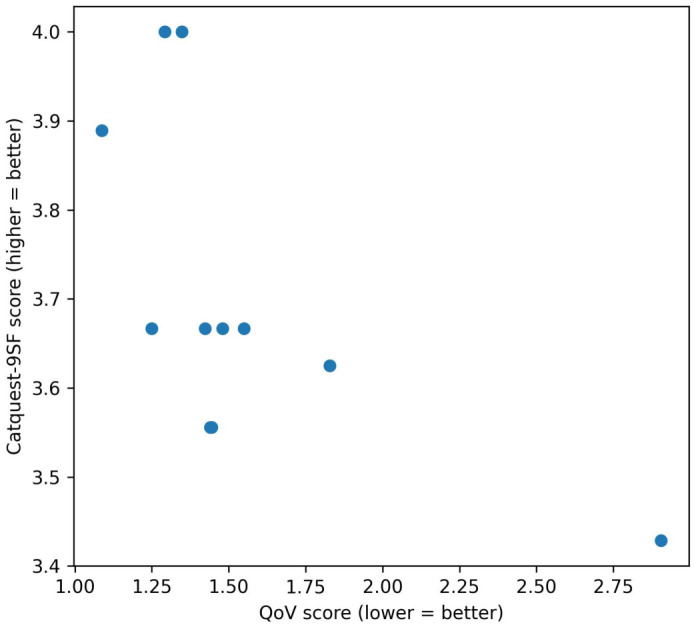
Relationship between patient-reported visual quality and satisfaction at two-year follow-up. Scatterplot showing the relationship between Quality of Vision (QoV) scores and Catquest-9SF scores in patients implanted with the diffractive IPCL. Each point represents one patient evaluated at two years postoperatively.

Patient-reported outcomes were analyzed at the patient level (*n* = 11). Overall, subjective visual quality and satisfaction were high. The distribution and relationship between QoV and Catquest-9SF scores are illustrated in Figure 3.

The QoV questionnaire showed a mean global score of 1.41 ± 0.43 (range: 1.06–2.63), indicating low frequency, intensity, and bother of visual symptoms. The Catquest-9SF demonstrated a mean score of 3.70 ± 0.18 (range: 3.43–4.00), consistent with high perceived visual function and independence.

Exploratory correlation analyses did not demonstrate significant associations between subjective quality of vision scores and objective functional or anatomical parameters, including the area under the defocus curve, scotopic pupil diameter, or postoperative vault.

### 3.6. Exploratory Associations Between Objective Performance, Anatomy, and PROMs

Exploratory analyses were conducted to investigate potential associations between objective visual performance metrics, anatomical positioning parameters, and patient-reported outcomes. In this cohort, no statistically significant correlations were identified between QoV scores and the mean area under the defocus curve (ρ = 0.26; *p* = 0.43), mean scotopic pupil diameter (ρ = 0.10; *p* = 0.76), or mean central vault (ρ = 0.01; *p* = 0.98).

Regarding the relationship between QoV and crystalline lens rise, although a moderate positive correlation was observed (ρ ≈ 0.64), it did not reach statistical significance (*p* ≈ 0.086). Considering the limited sample size, these findings should be interpreted as exploratory and hypothesis-generating rather than confirmatory.

Additional exploratory analyses evaluated potential relationships between lens parameters and postoperative vault. No significant correlation was found between absolute spherical power of the implanted IPCL and central vault (Spearman ρ = −0.03; *p* = 0.88). Likewise, vault values did not differ significantly according to near addition power (2.5 D vs. 3.0 D; ρ = −0.15; *p* = 0.51). Eyes implanted with toric IPCL models showed vault measurements comparable to those of non-toric lenses (*p* = 0.49).

These findings suggest that, within the range of lens parameters used in this cohort, postoperative vault appears to be primarily determined by individual anatomical factors rather than by lens optical design or refractive power. Given the limited sample size, all correlation analyses should be interpreted as exploratory and hypothesis-generating rather than confirmatory.

### 3.7. Environmental Evaluation

Environmental conditions during the implantation and follow-up period corresponded to a subtropical Atlantic coastal climate characterized by high annual solar radiation, persistent trade winds, and stable temperature and humidity profiles. Data obtained from the Spanish State Meteorological Agency (AEMET) at the Gran Canaria Airport meteorological station (Gando) demonstrated minimal interannual variability between 2020 and 2026, with mean annual temperatures ranging from 21.3 °C to 22.2 °C, relative humidity between 65% and 69%, and mean wind speeds of approximately 21–23 km/h.

Recurrent Saharan dust intrusions (“calima”), averaging approximately 18–27 days per year, were documented throughout the study period. These atmospheric events are known to increase light scattering and reduce contrast under real-world visual conditions. Mean solar radiation remained consistently high across all years analyzed, reflecting sustained exposure to outdoor photopic environments.

All patients resided in Las Palmas de Gran Canaria, an urban coastal setting with continuous exposure to marine aerosols, wind-driven particulate matter, and high ambient luminance. This geographic and climatic uniformity supports environmental homogeneity across the cohort during both implantation and long-term postoperative follow-up, providing a consistent real-world context for the interpretation of functional and patient-reported visual outcomes (Table 2).

## 4. Discussion

This study provides a comprehensive functional, anatomical, and contextual evaluation of a diffractive implantable phakic contact lens in presbyopic myopic patients with a two-year follow-up. In this age group, several surgical strategies can be considered for simultaneous correction of myopia and presbyopia, including excimer laser monovision, presbyopic corneal ablation profiles, or refractive lens exchange. However, these approaches may involve trade-offs such as reduced binocular visual quality, progressive near vision decline, or increased retinal detachment risk in highly myopic eyes. In this context, lens-preserving approaches such as diffractive phakic lenses may represent an attractive alternative. Beyond reporting visual acuity and anatomical safety, the present work contextualizes lens performance within a real-world high solar radiation Atlantic environment, offering an ecological perspective rarely addressed in refractive lens research.

### 4.1. Functional Performance Across Visual Distances

The monocular defocus curve demonstrated a broad plateau of high-quality visual performance from distance through intermediate and functional near ranges, with mean acuity remaining ≤0.14 logMAR up to −2.5 D of defocus. The gradual decline beyond −3.0 D reflects the expected optical behavior of a diffractive design targeting balanced performance across distances. Importantly, the functional range defined as ≤0.2 logMAR extended to approximately −3.0 D in half of the eyes, supporting effective visual function for common daily activities including intermediate and near tasks.

Earlier clinical experiences with diffractive IPCL designs have reported encouraging visual outcomes. Bianchi and Schmid et al. described favorable early distance and near performance, although detailed defocus curve characterization beyond the first postoperative year remains limited [7,8]. In the series reported by Bianchi, binocular defocus curves showed mean visual acuity values of approximately 0.08 logMAR at distance, 0.11 logMAR at −1.5 D, and 0.06 logMAR at −3.0 D at one year postoperatively [7]. Although direct comparison is limited by methodological differences, including binocular versus monocular testing, both studies demonstrate a broad plateau of functional vision spanning distance, intermediate, and near ranges. The present analysis confirms that this functional profile is maintained over time and complements previous observations by integrating anatomical stability and patient-reported outcomes.

Unlike conventional spectacle prescriptions, the addition power in diffractive phakic lenses may be selected with a forward-looking strategy, aiming to maximize functional longevity as presbyopia progresses. The individualized tolerance-based approach used in this study allowed selection of the highest comfortable addition while preserving distance visual quality.

The functional visual range observed in this cohort is consistent with the optical principles underlying pseudoaccommodation. Depth of field in presbyopic eyes can be enhanced by optical factors such as pupil diameter, residual astigmatism, and higher-order aberrations, as well as by diffractive optical designs that distribute light across multiple focal points [21,22,23]. This light distribution inherently involves a trade-off between image contrast and depth of focus, which is reflected in the defocus curve profile observed in multifocal optical systems [24]. However, such strategies may involve trade-offs, including dysphotopsia and contrast sensitivity reduction, commonly described in multifocal and extended-depth-of-field intraocular lenses.

From an optical perspective, diffractive designs redistribute incident light into multiple focal points, generating simultaneous retinal images at different vergences. This light-splitting mechanism inherently involves trade-offs between image contrast and depth of focus, which are reflected in the characteristic defocus curve profile. Recent optical modeling approaches, including Fourier-based analyses of multifocal phase profiles, have further demonstrated how diffractive structures can optimize energy distribution to extend functional vision while maintaining acceptable image quality across distances. Within this framework, the broad plateau observed in the present study likely reflects an effective balance between light distribution efficiency and neural tolerance to simultaneous vision.

It should be noted that defocus curves were obtained monocularly to isolate the optical performance of each eye under standardized conditions. However, real-world visual function is inherently binocular, and binocular summation may further enhance functional performance. Therefore, the present findings may underestimate the effective visual range experienced under daily viewing conditions.

### 4.2. Anatomical Stability and Safety Profile

At 2 years, central vault values remained within clinically accepted limits, with a median of 546 µm and no evidence of progressive anatomical instability. Pupillary diameters and crystalline lens rise measurements reflected physiological variability without apparent impact on global visual performance metrics. These findings support the long-term anatomical compatibility of the IPCL design in presbyopic myopic patients, for whom crystalline lens preservation is particularly desirable.

Safety remains a central concern in phakic intraocular lens implantation, especially regarding cataract formation and endothelial health [22,25,26,27,28,29]. In the present series, no cases of clinically significant cataract development were documented at two years of follow-up, and all eyes maintained clear crystalline lenses and corneas on slit-lamp examination. Notably, even in eyes with vault values outside the median range, no adverse anatomical or optical sequelae were observed, suggesting that moderate vault variability within clinically accepted limits may not necessarily translate into compromised safety in the short to medium term [11,27,29].

Although endothelial cell density and pachymetric changes were not systematically quantified at the two-year time point due to the retrospective design, detailed slit-lamp evaluation confirmed preserved corneal transparency and absence of clinically relevant corneal alterations in all eyes. Also, although dynamic vault behavior was not evaluated, previous studies have reported limited vault variability with IPCL designs. These findings align with previous reports demonstrating stable vault behavior and low incidence of visually significant cataract formation following IPCL implantation when appropriate vault is achieved and maintained [29,30]. Together, these findings suggest favorable short- to mid-term anatomical outcomes in this cohort; however, the absence of longitudinal endothelial cell density data and the limited sample size preclude definitive conclusions regarding long-term safety.

### 4.3. Patient-Reported Outcomes and Functional Perception

Patient-reported visual quality and satisfaction were favorable overall, with low QoV scores and high Catquest-9SF values across the cohort. The inverse relationship observed between QoV and Catquest scores supports internal consistency between perceived symptom burden and global functional satisfaction.

The use of two complementary patient-reported outcome measures warrants consideration. The QoV questionnaire primarily captures the frequency, severity, and bothersomeness of dysphotopsias and visual disturbances [17], whereas the Catquest-9SF evaluates broader aspects of visual disability, independence from spectacles, and overall satisfaction [18]. Previous literature has emphasized that visual symptoms and functional disability represent related but distinct dimensions of visual experience after refractive or presbyopia-correcting interventions [31,32,33,34]. Accordingly, combining symptom-specific and function-oriented PROMs provides a more comprehensive characterization of patient-perceived outcomes.

Importantly, exploratory analyses did not demonstrate strong associations between objective optical or anatomical metrics and subjective quality scores. This finding is consistent with prior reports in multifocal IOL and presbyopia-correcting lens literature, where patient satisfaction depends not only on measurable optical parameters but also on neural adaptation, expectation management, and real-world visual demands [31,32,33,34]. These results reinforce the concept that patient-perceived visual quality after presbyopia-correcting phakic lens implantation is multifactorial and cannot be fully explained by isolated anatomical or optical variables alone.

To our knowledge, the combined use of both QoV and Catquest-9SF questionnaires has not been routinely reported in studies evaluating posterior chamber phakic intraocular lenses, including spherical, toric, or diffractive designs. Most published series have focused primarily on objective visual and refractive outcomes, with limited integration of multidimensional patient-reported outcome measures. The present approach, incorporating both symptom-specific and function-oriented PROMs, therefore provides a more comprehensive understanding of patient-perceived visual performance and may contribute to a more patient-centered evaluation framework for presbyopia-correcting phakic lens implantation.

Interestingly, patient-reported dysphotopsia scores were low in this cohort despite the diffractive optical design. Although multifocal and extended-depth-of-field intraocular lenses are often associated with dysphotopsia phenomena and contrast sensitivity trade-offs, phakic diffractive designs may interact differently with the native crystalline lens and ocular aberrations, potentially modulating the subjective perception of photic phenomena [22]. The presence of the natural crystalline lens may also contribute to spectral filtering and modulation of light transmission, which could partially attenuate dysphotopsia perception compared with pseudophakic diffractive optics.

### 4.4. Environmental Context and Real-World Exposure

A distinctive feature of this study is the environmental context in which patients were evaluated, namely the Atlantic island setting of Gran Canaria. This region is characterized by sustained high solar radiation, persistent trade winds, marine aerosol exposure, and recurrent Saharan dust events (“calima”), creating a naturally demanding optical environment with elevated luminance, atmospheric light scattering, and frequent outdoor exposure. The island’s compact geography, with a maximum inland distance to the coastline of less than 50 km, results in continuous population exposure to maritime light reflection and coastal photopic conditions.

Despite these potentially challenging visual conditions, both functional performance and patient-reported outcomes remained stable and favorable within this cohort. This ecological consistency provides a real-world context for interpreting the findings, suggesting that diffractive IPCL performance can be consistently observed not only under controlled clinical conditions but also within this environmental setting.

Although the Canary Islands constitute an active volcanic archipelago and experienced a major eruption on La Palma in 2021, no volcanic eruptions or ash deposition events occurred on Gran Canaria during the study period. Atmospheric conditions affecting this cohort were therefore primarily driven by recurrent Saharan dust intrusions and marine aerosol exposure. While the present study was not specifically designed to evaluate environmental influences, the geographic and climatic characteristics of Gran Canaria may represent a relevant real-world context for assessing visual performance in presbyopia-correcting phakic lenses and deserve further investigation.

In subtropical coastal environments such as Gran Canaria, lifestyle patterns frequently include extended evening and nighttime outdoor activities. Under these conditions, dysphotopsia-related symptoms such as halos or glare could theoretically become more noticeable, particularly during episodes of Saharan dust intrusion (“calima”). Despite this potentially demanding visual context, patient-reported outcomes remained favorable.

From a broader perspective, these findings highlight the potential value of evaluating refractive technologies within real-world environmental contexts, where visual performance is ultimately experienced. High-luminance coastal environments and variable atmospheric transparency represent real-world visual stressors that may influence subjective and functional performance. While the present study was not designed to directly evaluate environmental effects, the consistent functional and patient-reported outcomes observed within this setting provide a descriptive real-world context for interpreting diffractive IPCL performance. These findings should therefore be understood as contextual rather than causal.

### 4.5. Limitations

This study is limited by its retrospective design and relatively small sample size, which restricts statistical power and generalizability. The retrospective nature may introduce selection bias and limits control over potential confounding factors despite standardized clinical assessment. Endothelial cell density and pachymetric measurements were not uniformly available at the two-year follow-up and were therefore not included in the quantitative analysis. This represents a major limitation in the assessment of long-term corneal safety.

Defocus curves were obtained monocularly, which may not fully reflect real-world binocular visual performance. The environmental assessment was descriptive and exploratory, and no direct measurements of visual performance under varying environmental conditions were performed. Consequently, causal relationships between environmental factors and visual outcomes cannot be established. Accordingly, findings should be interpreted within the context of an exploratory cohort.

## 5. Conclusions

In this exploratory cohort of presbyopic myopic patients, bilateral implantation of a diffractive IPCL was associated with stable anatomical positioning, a broad range of functional visual performance across distances, and favorable patient-reported outcomes at two years. Within a consistent real-world environmental context, these findings provide a descriptive framework for understanding diffractive IPCL performance, while larger prospective studies are warranted to confirm these observations.

## Figures and Tables

**Table 1:** Implanted Lens Optical Characteristics.

Value	Parameter
−7.16 ± 3.55 D	Mean spherical power
−14.0 to −2.5 D	Range spherical power
+2.66 ± 0.24 D	Mean addition
+2.5 to +3.0 D	Addition range
31.8%	Toric models

**Table 2:** Environmental and Atmospheric Optical Conditions in Gran Canaria During the Implantation and Follow-up Period (AEMET-Gando Station, 2020–2026).

Solar Radiation (kWh/m^2^/day)	Saharan Dust Events (Calima Days/Year)	Wind km/h	Humidity %	Mean Temp °C	Year
5.4	18	21	69	21.3	2020
5.5	22	22	68	21.5	2021
5.6	27	23	67	21.7	2022
5.7	19	22	66	22.0	2023
5.8	24	21	65	22.2	2024
5.7	21	22	66	22.1	2025
5.7	20	22	66	22.0	2026

## Data Availability

The data supporting the findings of this study are not publicly available due to local regulatory and ethical restrictions. Data may be made available from the corresponding author upon reasonable request and subject to institutional approval.

## Abbreviations

The following abbreviations are used in this manuscript:

AUC	area under the defocus curve
IPCL	implantable phakic contact lens
logMAR	logarithm of the minimum angle of resolution
PROMs	patient-reported outcome measures
QoV	Quality of Vision
